# Late diagnosis of Human Immunodeficiency Virus infection and associated factors*

**DOI:** 10.1590/1518-8345.4072.3342

**Published:** 2020-08-31

**Authors:** Luana Carla Santana Ribeiro, Maria Imaculada de Fátima Freitas, Unaí Tupinambás, Francisco Carlos Félix Lana

**Affiliations:** 1 Universidade Federal de Campina Grande, Centro de Educação e Saúde, Cuité, PB, Brazil.; 2 Universidade Federal de Minas Gerais, Escola de Enfermagem, Belo Horizonte, MG, Brazil.; 3 Universidade Federal de Minas Gerais, Faculdade de Medicina, Belo Horizonte, MG, Brazil.

**Keywords:** HIV, Acquired Immunodeficiency Syndrome, HIV Infections, Delayed Diagnosis, Early Diagnosis, Cross-Sectional Studies, HIV, Síndrome de Imunodeficiência Adquirida, Infecções por HIV, Diagnóstico Tardio, Diagnóstico Precoce, Estudos Transversais, VIH, Síndrome de Inmunodeficiencia Adquirida, Infecciones por VIH, Diagnóstico Tardío, Diagnóstico Precoz, Estudios Transversales

## Abstract

**Objective:**

to analyze the occurrence of late diagnosis of infection by the Human Immunodeficiency Virus and its associated factors.

**Method:**

this is an epidemiological, cross-sectional and analytical study, carried out with 369 people followed-up by Specialized Assistance Services, undergoing anti-retroviral treatment, and interviewed by means of a questionnaire. Univariate analysis was performed using Pearson’s chi-square test or Fisher’s exact test and Kruskall-Wallis test, and multivariate analysis using the ordinal logistic regression model of proportional odds.

**Results:**

the occurrence of 59.1% for late diagnosis of the infection was observed; the probability of later diagnosis is greater among people who have a steady partnership, when compared to those who do not; with increasing age, particularly above 35 years old; among those with lower schooling; for those who seek the health services to have an HIV test when they feel sick; and for those who test HIV less often or never do it after sex without a condom with a steady partner.

**Conclusion:**

the knowledge on the high proportion of late diagnosis and its associated factors verified in this study make the planning and implementation of new policies and strategies aimed at the timely diagnosis of the infection imperative.

## Abstract (pt)

**Objetivo:**

analisar a ocorrência de diagnóstico tardio de infecção pelo Vírus da Imunodeficiência Humana e seus fatores associados.

**Método:**

trata-se de estudo epidemiológico, transversal e analítico, realizado com 369 pessoas acompanhadas por Serviços de Assistência Especializada, em tratamento antirretroviral, entrevistadas por meio de questionário. Realizou-se análise univariada, utilizando-se os testes Qui-quadrado de Pearson ou exato de Fisher e o teste de Kruskall-Wallis, e análise multivariada, pelo modelo de regressão logística ordinal de chances proporcionais.

**Resultados:**

observou-se a ocorrência de 59,1% de diagnóstico tardio da infecção; a probabilidade de diagnóstico mais tardio é maior entre pessoas que têm parceria fixa, quando comparadas aos que não têm; com o aumento da idade, particularmente, acima dos 35 anos; entre os que possuem menor escolaridade; para os que buscam os serviços de saúde para realizar o teste de HIV quando se sentem doentes; para aqueles que fazem o teste de HIV com menor frequência ou nunca o fazem após relação sexual sem preservativo com parceria fixa.

**Conclusão:**

o conhecimento da elevada proporção de diagnóstico tardio e de seus fatores associados verificados neste estudo tornam imperiosos o planejamento e a implementação de novas políticas e estratégias que visem ao diagnóstico oportuno da infecção.

## Abstract (es)

**Objetivo:**

analizar la ocurrencia del diagnóstico tardío de infección por el Virus de Inmunodeficiencia Humana y sus factores asociados.

**Método:**

estudio epidemiológico, transversal y analítico, realizado con 369 personas que utilizan Servicios de Asistencia Especializada, en tratamiento antirretroviral, entrevistados a través de un cuestionario. El análisis univariado se realizó por medio de la prueba de chi-cuadrado de Pearson o la prueba exacta de Fisher y la prueba de Kruskall-Wallis, mientras que para el análisis multivariado se utilizó el modelo de regresión logística ordinal de probabilidades proporcionales.

**Resultados:**

se observó la ocurrencia de 59,1% de diagnóstico tardío de la infección; la probabilidad de un diagnóstico posterior es mayor entre las personas que tienen una pareja estable, en comparación con quienes no la tienen; con el aumento de la edad, particularmente, por encima de los 35 años; entre aquellos con índice más bajo de escolaridad; para aquellos que buscan servicios de salud para hacerse una prueba de VIH cuando se sienten enfermos; para quienes se realizan la prueba del VIH con menor frecuencia o nunca lo hacen después de tener relaciones sexuales sin condón con una pareja estable.

**Conclusión:**

el conocimiento de la alta proporción de diagnósticos tardíos y sus factores asociados verificados en este estudio hacen que la planificación e implementación de nuevas políticas y estrategias dirigidas al diagnóstico oportuno de la infección sea imperativa.

## Introduction

Since the beginning of the Human Immunodeficiency Virus (HIV) infection pandemic in the 1980s, Brazil has implemented a series of government and social measures to tackle the epidemic. In the last few decades, a significant decrease in AIDS morbidity and mortality has been observed in the country, due to the introduction of universal and free access to anti-retroviral therapy (ART), the harm reduction policy, the implementation of combined prevention strategies, the recommendation of treatment as prevention, and the wide range of diagnostic tests^(^ 1 ^-^ 3 ^)^. These strategies have jointly contributed to the increase in the life expectancy and quality of life of people living with HIV (PLHIV), to the decrease in hospital admissions due to the reduction of opportunistic infections, and to the reduction of HIV transmission^(^ 4 ^-^ 6 ^)^.

Despite the efforts and integrated actions of the governments, of civil society, of social movements, and of non-governmental organizations in tackling the AIDS epidemic, the tendency for its morbidity and mortality to decline, the expanded access to ART, and the technological advances in case management, this condition remains at the top of public health problems, affecting the population’s quality of life and impacting the economy and the social and family structures^(^ 7 ^-^ 8 ^)^.

The delay in the diagnosis and the consequent late assistance to PLHIV are some of the main concerns in combating the epidemic^(^ 7 ^,^ 9 ^)^. The early diagnosis, together with the immediate initiation of the treatment, brings irrefutable health benefits to PLHIV, due to its greater effectiveness in maintaining the immune status and reducing morbidity and mortality. It also contributes to its prevention, since the spread of the infection is avoided in a phase marked by high viral loads and high infectious potential, which also results in greater investment of time and resources by the health systems^(^ 10 ^-^ 12 ^)^.

Clinical and laboratory monitoring of HIV infection is performed by counting CD4+T lymphocytes (LT-CD4+) and viral load (VL). According to data from the Ministry of Health (MoH), in 2018 27% of PLHIV arrived at the health service with an HIV infection late diagnosis (LD), considering the CD4 count criterion below 200 cells/mm^3(^ 13 ^)^.

A research study carried out in Brazil, which used the criterion of late onset of clinical follow-up for asymptomatic patients with an LT-CD4+ count below 350 cells/mm^3^, in the 2003-2006 period, revealed that the prevalence of late onset was 58.6%, resulting in an increase of more than a third in the AIDS mortality rates. Another relevant conclusion of the study was that if all patients had started treatment in a timely manner, the decrease in AIDS mortality could have been 62.5% (against the 43.0% observed), between 1995 and 2006, increasing by 45.2%^(^ 14 ^)^ the effectiveness of the program to deal with this epidemic.

The prevalence of late diagnosis of HIV infection was also estimated in other countries, such as the United States, Australia, France, Italy, and Canada, which presented proportions of this event that varied from 8.8% in Canada, to 28.7% in the United States, considering as one of the criteria a CD4 count below 200 cells/mm^3(^ 12 ^)^. A research study involving 30,454 individuals, from 34 European countries, estimated the prevalence of late onset (which can express late diagnosis or late entry to care), in the period from 2010 to 2013, at 47.9%, using the as a parameter a CD4 count below 350 cells/mm^3^ or an AIDS diagnosis within 6 months after HIV diagnosis^(^ 15 ^)^.

Timely diagnosis of HIV infection and prompt care for diagnosed people are an important part of the strategies in most countries. It is noteworthy that PLHIV using ART, which maintain LT-CD4+ counts above 500 cells/mm^3^ and undetectable VLs, reach a level of life expectancy similar to that of the general population^(^ 16 ^)^.

In Brazil, despite efforts to control the HIV epidemic, they are focused on the early diagnosis of the infection, on the treatment of PLHIV, and on the implementation of combined prevention interventions^(^ 17 ^)^ and, despite the high rates of late diagnosis, there is a scarcity of studies on this issue which address its quantitative dimension, such as the occurrence of late diagnosis according to current parameters and associated factors.

Thus, the objective of this study was to analyze the occurrence of late diagnosis of infection by the Human Immunodeficiency Virus and its associated factors.

## Method

This is an excerpt from the quantitative axis of a research with a mixed approach, carried out in the state of Paraíba, in the Northeast of Brazil. It is an epidemiological, cross-sectional and analytical study that included the six Specialized Assistance Services (SASs) of the state, in activity in 2017.

The population consisted of all the adults living with HIV on ART, followed-up by the referred SASs. The sample was of the stratified probabilistic type and the following inclusion criteria were considered: PLHIV on ART, over 18 years of age and in outpatient follow-up at the SASs. The exclusion criteria were the following: people with HIV hospitalized during the data collection period; and those with some neurological or cognitive disability that made it impossible for them to participate in the interview.

To perform the sample calculation, the 95% confidence interval (CI) was considered, the proportion of late diagnosis was 60%(18-19), the maximum allowed error was 5% and the probability of sample loss was 10%, resulting in a sample of 369 people with HIV. As the sampling was stratified, the sample calculation was performed for each stratum, that is, for each SAS, proportionally to the population.

For data collection, a questionnaire was used designed for research purposes, applied with a face-to-face interview, containing dichotomous, categorical, and Likert scale questions. Data collection was carried out from April to May 2017, using primary sources of research - interviews with PLHIV followed-up in the SASs and data from medical records to avoid memory bias. The medical records were consulted to obtain the LT-CD4+ count and viral load at the time of diagnosis, the date of diagnosis of HIV infection, the date on which ART was started, and the presence of AIDS-indicative disease at the time of diagnosis.

In order to ensure the minimum sample size in the period of one month of data collection and to select the research participants, a probabilistic draw of the days for the collection in each SAS was carried out. The number of days to be drawn in each service was obtained by the ratio between the minimum sample size calculated for each SAS and the mean daily attendance for dispensing anti-retroviral drugs in each service. An additional day (backup) was drawn for collection in each service, assuming the occurrence of unforeseen events or difficulties in the dynamics of the services’ operation. Thus, single-stage cluster sampling, stratified by the health service, was used for the draw procedure, with the day being the primary sampling unit and the strata designed by the establishments.

During data collection, the planning of the days drawn was not carried out exactly, due to the service logistics in the services, which on some dates did not allow access to interview users. These days were replaced by other dates that made it possible to reach the sample size previously established. In addition, there was approximately 15% of refusals to participate in the study, a fact already expected due to the fear of the stigma experienced by many PLHIV. However, it is noteworthy that the interviews were conducted in places that guaranteed the privacy of the participants, which favored people’s adherence to participate in the study, reaching the calculated sample.

Regarding the variables used in the study, the time of diagnosis was considered as the outcome, obtained by the LT-CD4+ count proxy variable at the time of diagnosis^(^ 10 ^,^ 12 ^,^ 14 ^,^ 19 ^)^. This response variable was categorized as follows: timely diagnosis, characterized by an LT-CD4+ count equal to or greater than 350 cells/mm^3^; late diagnosis, defined by LT-CD+ count equal to or greater than 200 cells/mm^3^ and below 350 cells/mm^3^; and very late diagnosis, measured by LT-CD4+ count below 200 cells/mm^3^ or characteristic AIDS disease in the initial exam, regardless of the LT-CD4+ count. The exposure variables used in the study included sociodemographic variables, related to access to HIV diagnosis and to the sexuality of PLHIV.

Data was stored and analyzed in the SPSS software, version 21.0. First, the proportions of the timely, late, and very late diagnosis of HIV infection were estimated, based on the LT-CD4+ count proxy variable at the diagnosis time.

In the univariate analysis to assess the factors associated with the diagnosis, classified as very late, late, and timely, the Pearson’s chi-square or Fisher’s exact tests were used in the analysis of categorical variables, and the Kruskall-Wallis test in the analysis of the numerical variables. All the numerical variables showed an asymmetric distribution, according to the Kolmogorov-Smirnov normality test.

In the multivariate analysis, to assess the factors associated with late diagnosis, as it is an ordinal categorical outcome (very late, late and timely diagnosis), the ordinal logistic regression model of proportional odds was used^(^ 20 ^)^. In this study, the Odds Ratio (OR) represents the chance of a patient having a later diagnosis.

During the modeling process, all the variables with a p-value below 0.20, according to the univariate analysis, were included in the multivariate model. The model was produced in blocks, according to the classification of the groups of variables: Block 1 - Sociodemographic factors; Block 2 - Factors related to access to the services; Block 3 - Factors related to sexuality. The model performed in each block used the backward method to remove the variables and only variables with a significance level equal to or less than 5% remained. Then, the variables that remained in the final model of each block were included in a single model simultaneously and the backward modeling process was performed again, with only the significant variables remaining at the 5% level.

After adjusting the final model, the OR values were estimated, with respective 95% confidence intervals (95% CIs). It should be noted that the final model presented a good fit, according to the Deviance statistic (p-value = 0.955), and the assumption of parallel lines proved to be valid (p-value = 0.590).

In all the analyses, the confidence intervals had a 95% confidence level and the p-value < 0.05 decided to reject the null hypothesis in the statistical tests used in this work. Finally, the results found in line with the relevant literature were discussed.

In compliance with CNS Resolution No. 466/2012, the research was submitted to the appreciation of Research Ethics Committees, through *Plataforma Brasil*, and approved with the following Opinion numbers: 1,870,281 (UFMG), 1,932,530 (UFPB - HULW), and 1,973,626 (UFCG - HUAC).

## Results

Most of the study participants were male (cisgender men) (55.0%) and were in adulthood, and aged 25 to 49 years old (68.3%) at the time they were diagnosed with HIV. However, the percentage of HIV-positive young people aged up to 24 years old is noteworthy, which corresponded to 20.4% of the respondents. As for race or skin color, 257 (69.6%) of the survey participants declared themselves to be brown (55.0%) or black (14.6%). At the time of diagnosis, most of the respondents were married or in a stable relationship (50.9%) and had a low level of schooling (52.0%).

Regarding the “time of diagnosis” outcome, it was evidenced that 59,1% of the total of the interviewees were late diagnosed (18.2% with a T CD4+ lymphocyte count of 200-349 cells/mm^3^) or very late diagnosed (40.9% with a T CD4+ lymphocyte count below 200 cells/mm^3^), and 37.4% of them received the diagnosis in a timely manner, with an LT-CD4+ value corresponding to 350 cells/mm^3^ or more. At the time of diagnosis, the mean and median LT-CD4+ values of the research participants were 313.29 cells/mm^3^ and 253 cells/mm^3^, respectively. The lowest LT-CD4+ count recorded at the time of diagnosis was equal to 2 cells/mm^3^ and the highest count was 1,743 cells/mm^3^.

The results also showed that 238 (64.5%) of the total participants had a high VL at the time of diagnosis, with values above 10,000 copies/ml.

Table 1, based on univariate analysis, shows sociodemographic variables and their association with the diagnosis of HIV infection, classified as very late, late, and timely.

**Table 1:** Sociodemographic factors and association with HIV infection diagnosis. Paraíba, PB, Brazil, 2017 (n = 356)

Variable	Diagnosis			p-value
	Very late (n = 151)	Late (n = 67)	Timely (n = 138)	
**Gender**				
Female	58 (37.6%)	30 (19.5%)	66 (42.9%)	0.364*
Male	91 (46.9%)	34 (17.5%)	69 (35.6%)	
Trans-sexual/Transvestite	2 (28.6%)	2 (28.6%)	3 (42.8%)	
**Pregnancy**				
Yes	6 (13.6%)	11 (25.0%)	27 (61.4%)	**<0.001** ^†^
No	49 (47.5%)	18 (17.5%)	36 (35.0%)	
**Age in years old**				
Mean ± Standard Deviation	37.6 ± 10.9	35.9 ± 11.4	30.6 ± 10.9	**<0.001** ^‡^
Median (minimum - maximum)	37 (3 - 63)	35 (19 - 66)	28.5 (1 - 74)	
**Ethnicity**				
White	49 (49.0%)	16 (16.0%)	35 (35.0%)	0.219*
Black	16 (29.6%)	14 (25.9%)	24 (44.5%)	
Brown	84 (43.3%)	36 (18.6%)	74 (38.1%)	
Others	1 (16.7%	1 (16.7%)	4 (66.6%)	
**Marital status**				
No stcady partner	67 (39.0%)	27 (15.7%)	78 (45.3%)	**0.042** ^†^
With steady partner	84 (45.7%)	40 (21.7%)	60 (32.6%)	
**Religion**				
No religion	23 (33.3%)	19 (27.6%)	27 (39.1%)	0.037*
Catholic	93 (47.9%)	34 (17.6%)	67 (34.5%)	
Evangelic	30 (40.5%)	13 (17.6%)	31 (41.9%)	
Others	5 (26.3%)	1 (5.3%)	13 (68.4%)	
**Escholing in years**				
Mean ± Standard Deviation	7.4 ± 4.3	7.9 ± 4.3	9.5 ± 4.6	**<0.001** ^‡^
Median (minimum - maximum)	6 (0 - 20)	7 (0 - 20)	11 (0 - 23)	
**Affective sexual orientation**				
Heterosexual	109 (43.3%)	50 (19.8%)	93 (36.9%)	0.769*
Homosexual	31 (39.7%)	12 (15.4%)	35 (44.9%)	
Bisexual	11 (44.0%)	5 (20.0%)	9 (36.0%)	

Among the sociodemographic factors, there was an association with the diagnosis of HIV infection (p-value < 0.05): pregnancy when the infection was discovered, age, marital status, religion, and schooling. It appears that women in general were diagnosed later than pregnant women. The pregnancy variable was not included in the multivariate analysis as it is a condition that applies only to women of childbearing age and not to the general population investigated in the study.

Regarding age, the longer the life span, the later the diagnosis took place. The mean age of the participants who were diagnosed very late was 37.6 years and, of those diagnosed late, 35.9 years old.

People with a steady partnership were diagnosed later, as were those of the Catholic religion. In addition, it was shown that the lower the schooling level (in years of study), the later the diagnosis.

Table 2 shows the factors related to access to the health services and their association with HIV infection diagnosis.

**Table 2:** Factors related to access to the services and association with the diagnosis of HIV infection. Paraíba, PB, Brazil, 2017 (n = 356)

Variable	Diagnosis			p-value
	Very late (n = 151)	Late (n = 67)	Timely (n = 138)	
**Motivation of the person to seek the health service to perform the test HIV**				
Medical or prenatal indication	47 (35.3%)	31 (23.3%)	55 (41.4%)	**<0.001** *
For feeling sick	77 (73.4%)	14 (13.3%)	14 (13.3%)	
After sexual intercourse with a positive HIV partner or to know their serological status	23 (23.7%)	17 (17.5%)	57 (58.8%)	
After an unprotected relationship, blood donation, illnes of spouse or others	4 (19.1%)	5 (23.8%)	12 (57.1%)	
**First health service sought for HIV testing**				
BHU/FHU	24 (32.0%)	19 (25.3%)	32 (42.7%)	0.177*
Reference clinic, SAS or TCC	30 (41.6%)	11 (15.3%)	31 (43.1%)	
Public or Private Hospital	66 (50.7%)	21 (16.2%)	43 (33.1%)	
Private practice clinic, first-aid service or others	30 (38.5%)	16 (20.5%)	32 (41.0%)	
**Time to receive the result**				
Same day	72 (41.6%)	28 (16.2%)	73 (42.2%)	0.070*
Less than a week	35 (54.6%)	9 (14.1%)	20 (31.3%)	
More than a week	44 (37.3%)	30 (25.4%)	44 (37.3%)	
**Times they needed to go to the health service to find out that they had HIV/AIDS**				
Mean ± Standard Deviation	1.8 ± 2.5	2.6 ± 5.4	1.8 ± 2.1	0.088^†^
Median (minimum - maximum)	1 (1 - 20)	1 (1 - 44)	1 (1 - 22)	
**Health service that made the diagnosis**				
BHU/FHU	18 (32.7%)	15 (27.3%)	22 (40.0%)	0.100*
Reference clinic, SAS or TCC	35 (35.7%)	20 (20.4%)	43 (43.9%)	
Public or Private Hospital	75 (51.3%)	22 (15.1%)	49 (33.6%)	
Private clinic, first-aid service, LAB. P or others	22 (40.0%)	9 (16.4%)	24 (43.6%)	
**Distance from the house to the job place**				
Too far/Far away	86 (44.1%)	40 (20.5%)	69 (35.4%)	0.136*
Regular	25 (49.0%)	4 (7.9%)	22 (43.1%)	
Near/Very near	39 (35.8%)	23 (21.1%)	47 (43.1%)	
**Means of transportation used**				
Oublic transportation	76 (39.6%)	30 (15.6%)	86 (44.8%)	0.094*
Own car/motorcycle	33 (45.8%)	14 (19.5%)	25 (34.7%)	
Others	42 (45.7%)	23 (25.0%)	27 (29.3%)	

* Chi-square test;

† Kruskal Wallis.

The only variable that was statistically associated with a late diagnosis of HIV infection in relation to access to the health services, in the univariate analysis (p-value < 0.05), was the reason why they sought the health service to perform the HIV test (p-value < 0.001). It was verified that the diagnosis was later among those who sought the service because they felt sick.

Table 3 shows the variables related to the sexuality of the interviewed PLHIV that were associated with HIV infection diagnosis, in the univariate analysis.

**Table 3:** Factors related to sexuality and association with HIV infection diagnosis. Paraíba, PB, Brazil, 2017 (n = 356)

Variable	Diagnosis			p-value
	Very late (n = 151)	Late (n = 67)	Timely (n = 138)	
**Age when they had sex for the first time**				
Mean ± Standard Deviation	17 ± 4.7	15.9 ± 2.8	16.1 ± 3.8	0.348*
Median (minimum - maximum)	16 (9 - 47)	16 (8 - 22)	16 (8 - 36)	
**Used condom in the first intercourse**				
Yes	35 (36.5%)	17 (17.7%)	44 (45.8%)	0.256^†^
No	114 (44.2%)	50 (19.4%)	94 (36.4%)	
**Number of partners with whom they have already had sex**				
Up to 9	75 (37.1%)	41 (20.3%)	86 (42.6%)	0.083^†^
10 or more	73 (49.0%)	24 (16.1%)	52 (34.9%)	
**Frequency using the condom**				
Always or almost always	66 (43.7%)	24 (15.9%)	61 (40.4%)	0.736^†^
Sometimes	34 (39.5%)	20 (23.3%)	32 (37.2%)	
Never or almost never	51 (42.9%)	23 (19.3%)	45 (37.8%)	
**Frequency of performing the quick HIV test (after an unprotected relationship with a steady partner)**				
Always or almost always	4 (17.4%)	3 (13.0%)	16 (69.6%)	**0.028** ^‡^
Sometimes	3 (37.5%)	2 (25.0%)	3 (37.5%)	
Never or almost never	144 (44.3 %)	62 (19.1%)	119 (36.6%)	
**Frequency of quick HIV testing (after an unprotected intercourse with a casual partner)**				
Always or almost always	6 (33.3%)	2 (11.1%)	10 (55.6%)	0.367^‡^
Sometimes	4 (36.4%)	4 (36.4%)	3 (27.2%)	
Never or almost never	141 (43.1%)	61 (18.7%)	125 (38.2%)	
**Reasons that influenced for not using condoms** **Trusting the partner**				
Yes	120 (41.5%)	52 (18.0%)	117 (40.5%)	0.371^†^
No	31 (46.3%)	15 (22.4%)	21 (31.3%)	
**Having a steady partner**				
Yes	107 (42.3%)	45 (17.8%)	101 (39.9%)	0.659^†^
No	44 (42.7%)	22 (21.4%)	37 (35.9%)	
**Long-lasting relationship**				
Yes	107 (41.0%)	52 (19.9%)	102 (39.1%)	0.560^†^
No	44 (46.3%)	15 (15.8%)	36 (37.9%)	
**Religious reasons**				
Yes	14 (35.0%)	5 (12.5%)	21 (52.5%)	0.161^†^
No	137 (43.4%)	62 (19.6%)	117 (37.0%)	
**Decreases or takes away pleasure**				
Yes	67 (38.7%)	40 (23.1%)	66 (38.2%)	0.112^†^
No	84 (45.9%)	27 (14.8%)	72 (39.3%)	
**Partner refuses to use**				
Yes	63 (39.6%)	30 (18.9%)	66 (41.5%)	0.588^†^
No	88 (44.7%)	37 (18.8%)	72 (36.5%)	
**Dislikes using it**				
Yes	63 (43.2%)	25 (17.1%)	58 (39.7%)	0.812^†^
No	88 (41.9%)	42 (20.0%)	80 (38.1%)	
**Ashamed of suggesting its use to the partner**				
Yes	23 (65.7%)	3 (8.6%)	9 (25.7%)	**0.012** ^†^
No	128 (39.9%)	64 (19.9%)	129 (40.2%)	
**Not being able to buy**				
Yes	7 (41.2%)	5 (29.4%)	5 (29.4%)	0.492^‡^
No	144 (42.5%)	62 (18.3%)	133 (39.2%)	
**Others**				
Yes	3 (30.0%)	2 (20.0%)	5 (50.0%)	0.700^‡^
No	148 (42.,8%)	65 (18.8%)	133 (38.4%)	

* Kruskal Wallis;

† Chi-square test;

‡ Fisher's exact test

It was observed that the diagnosis was later among those who never tried to do the quick test after intercourse without a condom with a steady partner and who reported being ashamed to suggest the use of condoms to their partner (p-value < 0.05).

In the multivariate analysis, considering all the variables that remained in the final models of each block in a single model, the variables shown in Table 4 remained in the final model.

**Table 4:** Final model, according to ordinal logistic regression of proportional chances, for the assessment of the factors associated with HIV infection late diagnosis. Paraíba, PB, Brasil, 2017 (n = 356)

Variable	OR*	95% IC^†^		p-value
**Age (years old)**	1.03	1.01	1.06	0.001
**Marital status**				
No steady partner	1.00	-	-	-
With steady partner	1.58	1.01	2.46	0.045
**Schooling (years)**	0.94	0.89	0.99	0.016
**Motivation of the person to seek the health service to perform the HIV test**				
Medical or prenatal indication	1.00	-	-	-
Feeling sick	4.92	2.80	8.64	<0.001
After sexual intercourse with an HIV positive partner or to know their HIV status	0.60	0.35	1.02	0.060
After an unprotected relationship, blood donation, illness of spouse or others	0.52	0.20	1.34	0.176
**Frequency of quick HIV testing (after an unprotected intercourse with a steady partner)**				
Always or almost always	1.00	-	-	-
Sometimes	3.33	1.26	8.84	0.016
Never or almost never	6.40	1.23	33.25	0.027

* OR - Odds Ratio;

† 95% CI - 95% Confidence Interval; Deviance Statistics p-value = 0.955; Parallel Lines Test p-value = 0.590

According to these results, the following factors (p-values < 0.05), associated with HIV infection late diagnosis, remained in the final model: age, marital status, schooling, reason for which they sought the health service to perform the HIV test, and frequency of the quick HIV test after an unprotected relationship with a steady partner.

The results of the model indicate that, with the increase of one year old in age, the chance of a later diagnosis increases 1.03 times (or 3%), and can vary between 1.01 and 1.06 with 95% confidence. People with a steady affective partner have 1.58 times (or 58%) more chances of a later diagnosis than those who do not have a steady partner (95% CI = 1.01 - 2.46). The increase in schooling was associated with a lower chance of later diagnosis (OR = 0.94; 95% CI = 0.89 - 0.99).

In addition, HIV-positive individuals who reported having sought the health service to perform the HIV test because they feel sick are 4.92 times more likely to be diagnosed later than those who sought the service by medical indication or due to the performance of prenatal care (95% CI = 2.80 - 8.64).

Finally, the lower the frequency of quick HIV testing after unprotected sex with a steady partner, the greater the chance of a later diagnosis of the infection. Among those who never undergo screening, the chance of later diagnosis rises to 6.40 times (95% CI = 1.23 - 33.25).

## Discussion

The high occurrence of late or very late HIV infection diagnosis (59.1%) found portrays an alarming reality, with higher data than the national ones, in which the percentage of 42% of people diagnosed with a lower LT-CD4+ count was identified at 350 cells/mm^3^ in 2015^(^ 18 ^)^.

However, the percentage of delayed diagnosis of HIV infection was similar to results found in other developing countries, such as China, which corresponded to 72.6% in the period from 2009 to 2010^(^ 9 ^)^, Ethiopia, with 68.8% of delayed diagnosis in 2014^(^ 21 ^)^, and Mexico, with a late diagnosis prevalence of 49% in the period from 2008 to 2013^(^ 22 ^)^. However, these surveys used heterogeneous criteria to define a delay in the diagnosis or late onset, which non-standardizes the results and hinders the comparison among the countries.

In Brazil, research studies on late onset for the care with HIV infection published from 2011 to 2016 estimated prevalence rates ranging from 52.5% to 69.8%^(^ 14 ^,^ 23 ^-^ 26 ^)^. These results are below what is expected for consolidated public health programs for PLHIV, such as that of Brazil^(^ 24 ^)^.

Like Brazil, most of the countries in Latin America face a concentrated epidemic, with a large number of people still undiagnosed and a high prevalence of late diagnosis and, consequently, late onset of ART^(^ 27 ^)^. Late diagnosis is a continuous, worrying, and serious challenge for the control of the AIDS pandemic, as it is directly related to higher rates of morbidity and mortality^(^ 28 ^-^ 29 ^)^ and to the need for greater investment by the health systems^(^ 10 ^,^ 30 ^)^.

The gender variable was not a factor associated with late or very late diagnosis in this study, unlike other studies, which reported that men are more likely to have a LD of HIV infection or advanced disease when compared to women^(^ 15 ^,^ 31 ^-^ 32 ^)^. However, it was evident in the univariate analysis that women in general were diagnosed later than pregnant women. An equivalent result was found in a survey conducted in the Northeast of Brazil, which found that the prevalence of late onset of HIV, between 2007 and 2009, was twice as high among men (55.4%) and among women who did not become pregnant (56.0%), when compared to the prevalence in pregnant women (21.1%)^(^ 23 ^)^. Several studies have described similar percentages of LD among men and women, and it is relevant to consider pregnancy as a possible confounding variable, since the request for HIV testing is a prenatal routine and favors the timely diagnosis in pregnant women^(^ 12 ^,^ 23 ^)^.

As for the age variable, the longer the life span, the later the diagnosis. One of the aspects that may be related is the delay in carrying out HIV screening after unprotected sex, resulting in the discovery of the infection long after exposure to the virus and, thus, at an older age. A number of research studies indicate that older people are more likely to be diagnosed late, especially those aged 50 years old or older, which can be explained by the decreased risk perception and the subsequent reduction in the frequency of diagnostic tests in this group^(^ 25 ^,^ 29 ^,^ 32 ^-^ 34 ^)^.

Marital status is also a determining factor for LD. Being in a conjugal relationship can cause people to have an attitude of trust and a subsequent practice of sexual intercourse without a condom, due to the denial of the risk of becoming infected. Generally, the practice of protected sexual relations is limited to the beginning of the relationships and, as time goes by, the ability to negotiate condom use decreases, as does the “purification” of the partner, leading to abandoning the condom^(^ 35 ^)^. This practice happens since the beginning of sexual life in adolescence^(^ 36 ^)^.

In a study conducted in Turkey, the late onset for HIV care measures was more likely among married patients, diagnosed between 2003 and 2016. We emphasize the relationship of these results with the low perception of risk or its denial in these groups and the consequent failure to carry out diagnostic tests, in addition to the fact that, since married people are apparently considered to have a reduced risk of being infected, the physicians do not usually prescribe HIV tests^(^ 37 ^)^.

The main reasons for LD are the absence or low perception on the risk on the part of vulnerable people^(^ 38 ^-^ 39 ^)^. Generally, people with a steady affective partnership, due to the low perception of risk^(^ 37 ^)^, use condoms less frequently during sexual intercourse, which makes them vulnerable to HIV^(^ 40 ^)^ and to late diagnosis.

The increase in schooling associated with a lower chance for later diagnosis was also evidenced in other studies^(^ 26 ^,^ 29 ^,^ 38 ^,^ 41 ^-^ 42 ^)^. This direct relationship between low schooling and increased risk for LD is probably due to greater difficulty in the access to information on means of prevention and on the diagnosis, and to the consequent low level of knowledge about HIV/AIDS. A research study carried out in Ethiopia showed an association between educational status and knowledge about HIV/AIDS with the delay in diagnosing the infection. Having a college degree decreased the likelihood of people being tested late, and those with a high level of knowledge were 74% less likely to be diagnosed late, when compared to those with a low level of knowledge^(^ 42 ^)^.

The motivation for HIV screening and the frequency of testing after sexual intercourse with a steady partner were associated with the occurrence of the infection LD. Those individuals who were motivated to take the test for feeling sick, and those who took the test occasionally, almost never or never had a better chance of being diagnosed late. Periodic screening for the virus is related to people’s perception of the health-disease process and of their vulnerability to HIV, as well as to their access to the health services that offer the test^(^ 39 ^,^ 42 ^)^.

The fact that the main motivation for taking the test was feeling sick, among those who were diagnosed later, reveals that the conceptions that people build throughout life, about their health and illness, influence their actions to guarantee their own well-being. It can be said that people diagnosed with more delay are generally those who adopt health practices that are strictly aimed at curing their illnesses, which does not exclude the influence of access barriers, including for performing the test. These individuals have problems with access to cultural goods and social development, with no possibility of reconstructing ways of thinking and acting in relation to effective prevention in order not to get sick^(^ 43 ^)^.

A number of research studies show that people do not consider having an HIV test because they are apparently healthy^(^ 38 ^,^ 42 ^)^. In Brazil, as the epidemic is concentrated, people are referred for HIV screening usually only when they have symptoms suggestive of infection or when they exhibit certain behaviors that increase the risk of transmitting the virus. Thus, screening moves away from a risk-focused strategy, as it disregards those individuals who do not openly express attitudes that are generally associated with an increased risk of contamination^(^ 26 ^)^.

Therefore, recommendations are proposed to change the panorama of the LD of HIV infection in Brazil, such as: expanding the offer of HIV testing to all Family Health Units and encouraging annual screening of the virus for all people who have unprotected sex; discussing the problem of the LD of HIV infection in academic spaces, in an interdisciplinary way, aiming at the formulation of new coping strategies; and fostering the scientific production on the subject in the country, which still requires more studies focused on the different realities of the states and the regions.

As limitations of the research, the following are pointed out: the inexistence or deficiency of data registration in some medical records regarding the LT-CD4+ count and viral load at the time of diagnosis, the dates of diagnosis of HIV infection and of ART onset, and the presence of an AIDS indicative disease at the time of diagnosis. However, a 10% loss was foreseen in the sample calculation; therefore, these difficulties in obtaining the data did not compromise the reliability of the findings.

## Conclusion

The high occurrence of late diagnosis for this infection is predominantly associated with sociodemographic and sexual aspects, without disregarding factors related to access to the health services. The likelihood of a later diagnosis is greater as age increases, among people who have a steady partnership and lower schooling, in individuals who sought the health service to perform the HIV test because they felt sick, and among those who never or almost never performed the quick test after an unprotected sexual intercourse with a steady partner.

It should be noted that the analysis was carried out in a broad and in-depth manner with primary sources of investigation, addressing, in addition to sociodemographic variables, factors related to sexuality and access to the health services, which strengthens the reliability and relevance of the findings. The submitted results are expected to contribute to the design of new strategies and policies aimed at the timely diagnosis of the infection. To expand the investigation of this problem, it is suggested that new research studies be carried out, from the perspective of health professionals and managers at all levels of care in the Unified Health System.
